# Esophagitis Dissecans Superficialis: Malign Appearance of a Benign Pathology

**DOI:** 10.7759/cureus.8475

**Published:** 2020-06-06

**Authors:** Venkata R Rokkam, Avin Aggarwal, Sasha Taleban

**Affiliations:** 1 Inpatient Medicine, Banner University Medical Center, Tucson, USA; 2 Gastroenterology, Banner University Medical Center, Tucson, USA

**Keywords:** endoscopy, esophagus, benign, esophagitis, sloughing mucosa

## Abstract

Esophagitis dissecans superficialis (EDS), also known as sloughing esophagitis, is a very rare condition and may affect the whole esophagus, resulting in complete sloughing of the mucous membrane. EDS has been associated with various medications and dermatological conditions. In our case, EDS was suspected secondary to methotrexate treatment in a patient with Crohn's disease, although the definitive etiology remains unknown. It is very important for physicians to recognize the endoscopic appearance of EDS to provide appropriate clinical management and differentiate it from other esophageal disorders.

## Introduction

Esophagitis dissecans superficialis (EDS) is a rare endoscopic finding characterized by sloughing of large fragments of the esophageal squamous mucosa as strips and patches. To date, there have been a few reported cases of these abnormal endoscopic findings in the published literature, and the etiology and pathogenesis were unclear [1]. The patients present with varying symptoms such as dysphagia, heartburn, odynophagia, regurgitation, dyspepsia, upper gastrointestinal bleeding, anemia, and weight loss [2]. In our case, EDS was suspected secondary to methotrexate, although the definitive etiology remains unknown.

## Case presentation

**Figure 1:**
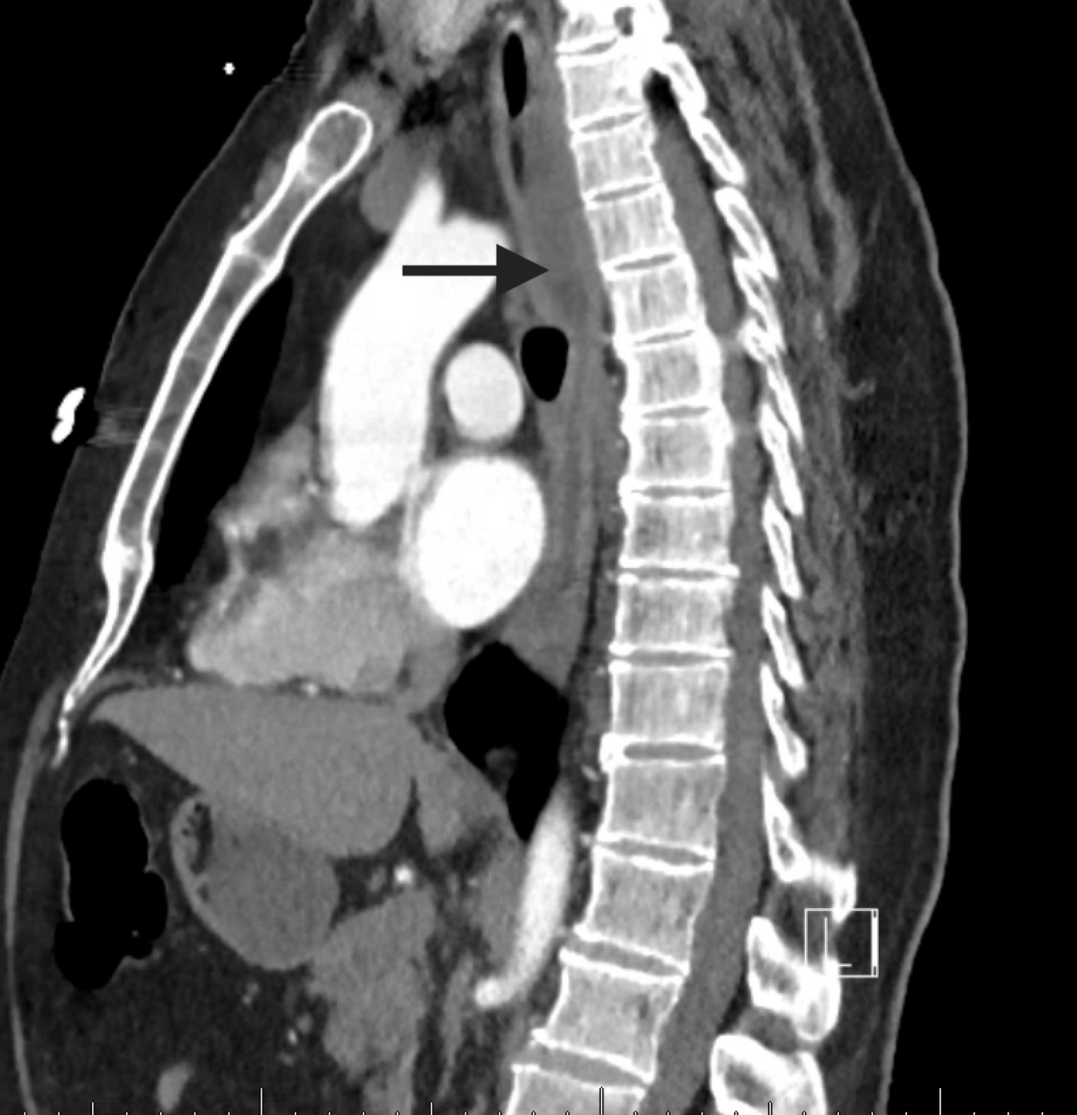
CT of the chest showing diffuse enlargement of the esophagus with small air-fluid level

**Figure 2:**
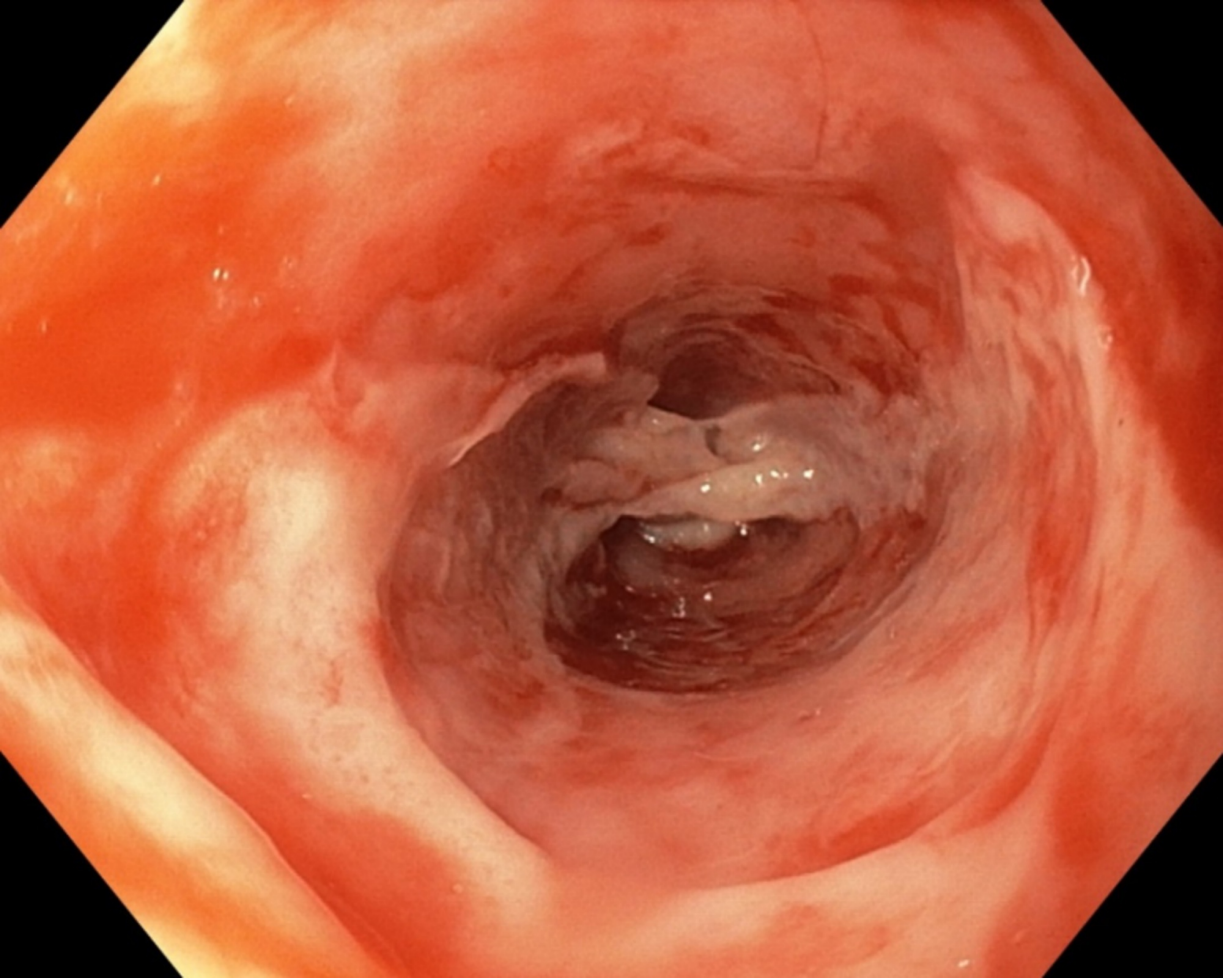
Image showing entire esophageal involvement on upper endoscopy

**Figure 3:**
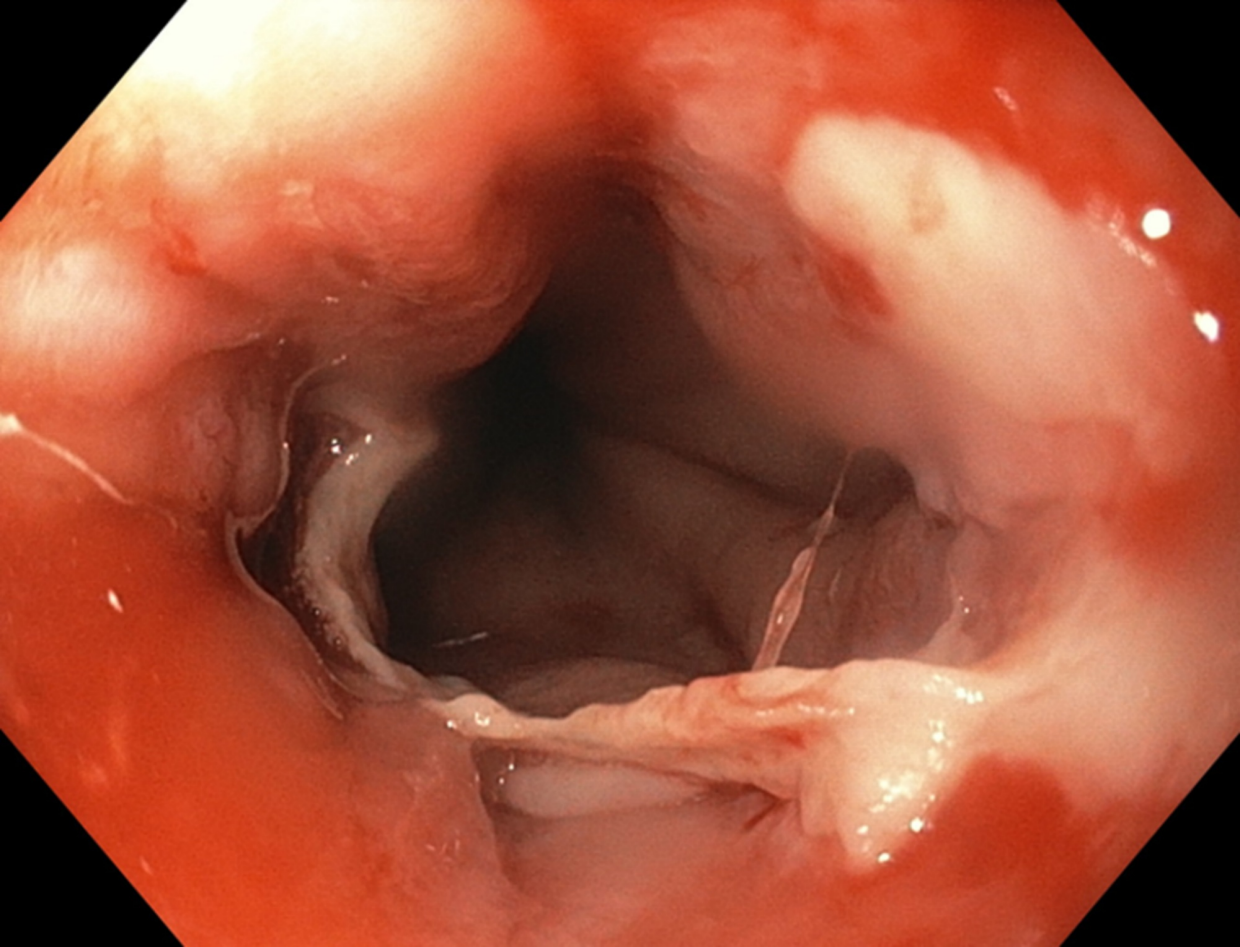
Image showing sloughing of several fragments of mucosa on upper endoscopy

A 57-year-old male with Crohn's disease on methotrexate, folic acid, and infliximab, and with a history of primary sclerosing cholangitis presented with an episode of hematemesis and acute onset odynophagia. He reported no abdominal pain, fever, weight loss, or the use of non-steroidal anti-inflammatory drugs. There was no prior history of upper gastrointestinal bleeding. He denied alcohol, tobacco, or illicit substance usage. Upon initial evaluation, he was found to be severely anemic with a hemoglobin level of 6.7 g/dL. Remaining laboratory values including complete metabolic panel, PT/INR (prothrombin time/international normalized ratio), and platelet count were within normal limits. Physical examination was unremarkable. CT scan of his chest showed diffuse thickening of the esophagus (Figure 1). Subsequent upper endoscopy was performed with esophageal findings (Figures 2, 3).

Endoscopy shows severe esophagitis with sloughing of the several fragments of mucosa along the length of the esophagus (Figure 2). The underlying area beneath the denuded mucosa is hyperemic and oozing blood in some areas. There is an appearance of white sloughing mucosa (Figure 3). These endoscopic findings are consistent with EDS. To avoid any bleeding, biopsy was not performed.

## Discussion

EDS is a rare endoscopic finding characterized by sloughing of large fragments of the esophageal squamous mucosa as strips and patches [3]. Although an association with medications (bisphosphonates/NSAIDs), desquamating disorders, particularly pemphigus vulgaris, heavy smoking, and physical trauma has been reported, the pathogenesis of EDS remains unexplained [4]. Endoscopy can help differentiate EDS from squamous cell carcinoma, candidal esophagitis, and peptic esophagitis. EDS is a benign condition that resolves without lasting esophageal pathology. Sometimes, the patient can develop esophageal casts that cause obstructive symptoms in the esophageal lumen [5]. The clinical course of EDS depends on the patient's underlying medical disease and is usually self-limited. There is no recommendation for a specific therapy or follow-up. Mucosal healing can be achieved through a combination of acid suppression and discontinuation of the precipitating factors and medications [6,7].

There has been a single reported case of EDS related to severe methotrexate toxicity with folate deficiency and pancytopenia, which improved after rescue folinic acid and discontinuation of methotrexate [6,7]. Our patient was on low-dose weekly methotrexate (7.5 mg) along with folic acid supplementation started around nine months prior to the presentation. He did not have pancytopenia or other evidence to suggest methotrexate toxicity. Although it is possible that our patient's presentation could be related to methotrexate, the definitive etiology remains unknown. The remainder of the hospital course remained uneventful without any further episodes of hematemesis and with a stable hemoglobin level. He was discharged on oral pantoprazole and sucralfate suspension. We recommended repeat upper endoscopy in eight weeks to check healing.

## Conclusions

Although it is possible that our patient's presentation could be related to methotrexate, the definitive etiology remains unknown. His remainder of the hospital course remained uneventful without any further episodes of hematemesis and with a stable hemoglobin level. Patient symptoms gradually improved with oral pantoprazole and sucralfate suspension. Though EDS appears malign on endoscopy, it is usually benign desquamative esophagitis. While EDS is a rare finding, it is very important that physicians recognize its endoscopic appearance to provide appropriate clinical management and relief of symptoms.
